# 1-(Prop-2-en-1-yl)-3-[(trimethyl­sil­yl)meth­yl]benzimidazolium bromide monohydrate

**DOI:** 10.1107/S1600536810033015

**Published:** 2010-08-25

**Authors:** Zeliha Baktır, Mehmet Akkurt, Nihat Şireci, Hasan Küçükbay

**Affiliations:** a Department of Physics, Faculty of Sciences, Erciyes University, 38039 Kayseri, Turkey; b Department of Chemistry, Faculty of Arts and Sciences, Adıyaman University, 02040 Adıyaman, Turkey; c Department of Chemistry, Faculty of Arts and Sciences, Ínönü University, 44280 Malatya, Turkey

## Abstract

In the title compound, C_14_H_21_N_2_Si^+^·Br^−^·H_2_O, the benzimidazole ring system is almost planar [maximum deviation = 0.021 (2) Å]. In the crystal, O—H⋯Br and C—H⋯O hydrogen bonds link the ions *via* the O atoms of the water mol­ecules. In addition, there are π–π stacking inter­actions between the centroids of the benzene and imidazole rings of the benzimidazole ring system [centroid–centroid distances = 3.521 (3) and 3.575 (2) Å].

## Related literature

For the anti­tumour activity of alkyl­silyl-substituted benzimidazole derivatives, see: Kleemann *et al.* (2009 ▶); Lukevics *et al.* (2001 ▶); Ignatovich *et al.* (2010 ▶). For the pharmacological activity of benzimidazole compounds, see: Singh & Lown (2000 ▶); Huang *et al.* (2006 ▶); Turner & Denny (1996 ▶); Galal *et al.* (2009 ▶); Küçükbay *et al.* (2003 ▶, 2004 ▶, 2009 ▶, 2010*a* ▶,*b* ▶); Şireci *et al.* (2010 ▶); Yılmaz & Küçükbay (2009 ▶); Yılmaz *et al.* (2010 ▶). For the structures of similar benzimidazole derivatives, see: Akkurt *et al.* (2008 ▶, 2010*a* ▶,*b* ▶); Yıldırım *et al.* (2006 ▶). For π–π inter­actions, see: Janiak (2000 ▶).
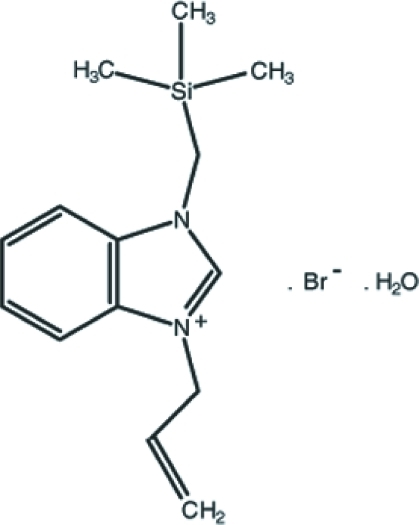


## Experimental

### 

#### Crystal data

C_14_H_21_N_2_Si^+^·Br^−^·H_2_O
*M* *_r_* = 343.33
Triclinic, 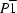

*a* = 8.9063 (2) Å
*b* = 10.4720 (2) Å
*c* = 10.9439 (3) Å
α = 66.542 (4)°
β = 71.479 (4)°
γ = 80.625 (5)°
*V* = 887.07 (5) Å^3^
*Z* = 2
Mo *K*α radiation
μ = 2.38 mm^−1^
*T* = 294 K
0.20 × 0.20 × 0.20 mm

#### Data collection

Rigaku R-AXIS RAPID-S diffractometer
Absorption correction: multi-scan (*SORTAV*; Blessing, 1995 ▶) *T*_min_ = 0.647, *T*_max_ = 0.647
18957 measured reflections
3618 independent reflections
2202 reflections with *I* > 2σ(*I*)
*R*_int_ = 0.073

#### Refinement

*R*[*F* ^2^ > 2σ(*F* ^2^)] = 0.049
*wR*(*F* ^2^) = 0.121
*S* = 1.02
3618 reflections
175 parameters
3 restraints
H-atom parameters constrained
Δρ_max_ = 0.30 e Å^−3^
Δρ_min_ = −0.35 e Å^−3^

### 

Data collection: *CrystalClear* (Rigaku/MSC, 2005 ▶); cell refinement: *CrystalClear*; data reduction: *CrystalClear*; program(s) used to solve structure: *SIR97* (Altomare *et al.*, 1999 ▶); program(s) used to refine structure: *SHELXL97* (Sheldrick, 2008 ▶); molecular graphics: *ORTEP-3 for Windows* (Farrugia, 1997 ▶) and *PLATON* (Spek, 2009 ▶); software used to prepare material for publication: *WinGX* (Farrugia, 1999 ▶).

## Supplementary Material

Crystal structure: contains datablocks global, I. DOI: 10.1107/S1600536810033015/dn2597sup1.cif

Structure factors: contains datablocks I. DOI: 10.1107/S1600536810033015/dn2597Isup2.hkl

Additional supplementary materials:  crystallographic information; 3D view; checkCIF report

## Figures and Tables

**Table 1:** Hydrogen-bond geometry (Å, °)

*D*—H⋯*A*	*D*—H	H⋯*A*	*D*⋯*A*	*D*—H⋯*A*
C7—H7⋯O1*W*	0.93	2.46	3.351 (5)	161
O1*W*—H1*W*⋯Br1	0.85	2.62	3.445 (4)	165
O1*W*—H2*W*⋯Br1^i^	0.85	2.59	3.360 (4)	152

Symmetry code: (i) 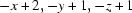.

**Fig. 1.**
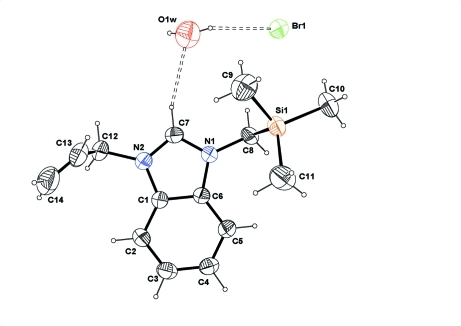
View of the title molecule with the atom numbering scheme. Displacement ellipsoids for non-H atoms are drawn at the 30% probability level. H atoms are represented as small spheres of arbitrary radii. Hydrogen bonds are shown as dashed lines.

**Fig. 2.**
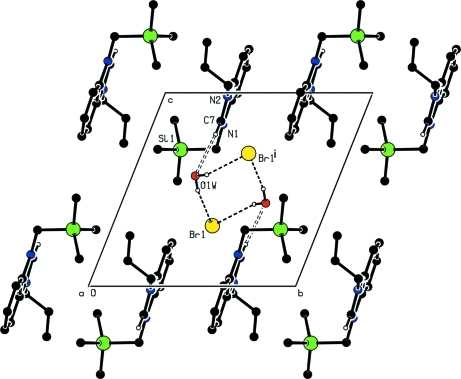
Packing view of the title compound with O–H···Br and C—H···O hydrogen bonds viewed down the a axis. H atoms not involved in hydrogen bondings are omitted for the sake of clarity. [Symmetry code: (i) -x+2, -y+1, -z+1]

## supplementary crystallographic information

### Comment

Heterocycles are important building blocks for the construction of anticancer drugs (Kleemann *et al.*, 2009). For example, alkylsilyl substituted benzimidazole derivatives have been reported to possess important antitumour activity (Lukevics *et al.*, 2001; Ignatovich *et al.*, 2010). Since, benzimidazole compounds have been found to have a broad range of pharmacological activity, many research groups as well as our group have been interested in these type of heterocyclic compounds (Singh & Lown, 2000; Huang *et al.*, 2006; Turner & Denny, 1996; Galal *et al.*, 2009; Küçükbay *et al.*, 2003, 2004, 2009, 2010*a*,*b*; Şireci *et al.*, 2010; Yılmaz *et al.*, 2009, 2010;). We have synthesized and investigated the crystal structures of many benzimidazole derivatives (Akkurt *et al.*, 2008, 2010*a*,*b*; Yıldırım *et al.*, 2006). Herein we report the synthesis and structure of the title compound, (I), 1-(prop-2-ene-1-yl)-3-[(trimethylsilyl)methyl]benzimidazolium bromide monohydrate.

The benzimidazole ring system (N1/N2/C1–C7) in the title molecule (I) (Fig. 1) is almost planar with a maximum deviation of 0.021 (2)Å for C1 atom. The bond lengths and angles in (I) are compatible with those found for similar compounds (Akkurt *et al.*, 2008, 2010*a*,*b*). The average Si—C bond length is 1.857 (5) Å. The angles around the Si atoms with a distorted tetrahedral geometry vary from 105.9 (2)° to 111.7 (3)°.

O–H···Br and C—H···O hydrogen bonds link the molecules (Table 1 and Fig 2). In the crystal structure, the benzene (C1–C6) and imidazole (N1/N2/C1/C6/C7) rings of the benzimidazole ring system form π-π stacking interactions with each other (Janiak, 2000) (Table 2).

### Experimental

A mixture of 1-(trimethylsilylmethyl)benzimidazole (1.02 g, 4.99 mmol) and allyl bromide (0.5 ml, 5,78 mmol) in dimethylformamide (5 ml) was refluxed for 3 h. The mixture was then cooled and the volatiles were removed under vacuum. The residue was crystallized from a dimethylformamide/ethanol (1:1). White crystals of the title compound (1.29 g, 79%) were obtained, m.p.: 394–395 °K; υ_(CN)_= 1552 cm^-1^. Anal. found: C 48.67, H 6.72, N 8.11%. Calculated for C_14_H_23_BrN_2_OSi: C 48.98, H 6.75, N 8.16%. ^1^H NMR (δ, DMSO-d_6_): 9.70 (s, 1H, NCHN), 8.14 - 7.67 (m, 4H, C_6_H_4_), 6.12 (m, 1H, CH allyl), 5.37 (m, 2H, CH_2_ allyl), 5.22 (d, 2H, CH_2_ allyl, *J*= 5.7 Hz), 4.26 (s, 2H, CH_2_Si) and 0.11 [s, 9H, (CH_3_)_3_Si]. ^13^C NMR (δ, DMSO-d_6_): 141.6 (NCHN), 132.5, 131.9, 131.3, 127.0, 126.8 and 120.4 (C_6_H_4_), 114.5 (CH allyl), 114.2 (CH_2_ allyl), 49.2 (CH_2_ allyl), 38.4 (CH_2_Si) and -2.2 [(CH_3_)_3_Si].

### Refinement

The hydrogen atoms on the water molecule were located from a difference Fourier map and refined with distance restraints of O—H = 0.85 (1) Å and H···H = 1.39 (1) Å, and with *U*_iso_(H) = 1.5 *U*_eq_(O). In the last steps of refinement, they were treated as riding on the parent O atom. The other H atoms were positioned geometrically, with C—H = 0.93–0.97 Å, and refined as riding with *U*_iso_(H) = 1.2 or 1.5 *U*_eq_(C).

### Crystal data

**Table:**

C_14_H_21_N_2_Si^+^·Br^−^·H_2_O	*Z* = 2
*M**_r_* = 343.33	*F*(000) = 356
Triclinic, *P*1	*D*_x_ = 1.285 Mg m^−^^3^
Hall symbol: -P 1	Mo *K*α radiation, λ = 0.71073 Å
*a* = 8.9063 (2) Å	Cell parameters from 3185 reflections
*b* = 10.4720 (2) Å	θ = 2.4–26.4°
*c* = 10.9439 (3) Å	µ = 2.38 mm^−^^1^
α = 66.542 (4)°	*T* = 294 K
β = 71.479 (4)°	Block, white
γ = 80.625 (5)°	0.20 × 0.20 × 0.20 mm
*V* = 887.07 (5) Å^3^	

### Data collection

**Table:**

Rigaku R-AXIS RAPID-S diffractometer	3618 independent reflections
Radiation source: Sealed Tube	2202 reflections with *I* > 2σ(*I*)
Graphite Monochromator	*R*_int_ = 0.073
Detector resolution: 10.0000 pixels mm^-1^	θ_max_ = 26.4°, θ_min_ = 2.4°
dtprofit.ref scans	*h* = −11→9
Absorption correction: multi-scan (*SORTAV*; Blessing, 1995)	*k* = −13→13
*T*_min_ = 0.647, *T*_max_ = 0.647	*l* = −13→13
18957 measured reflections	

### Refinement

**Table:**

Refinement on *F*^2^	Primary atom site location: structure-invariant direct methods
Least-squares matrix: full	Secondary atom site location: difference Fourier map
*R*[*F*^2^ > 2σ(*F*^2^)] = 0.049	Hydrogen site location: inferred from neighbouring sites
*wR*(*F*^2^) = 0.121	H-atom parameters constrained
*S* = 1.02	*w* = 1/[σ^2^(*F*_o_^2^) + (0.0356*P*)^2^ + 0.3659*P*] where *P* = (*F*_o_^2^ + 2*F*_c_^2^)/3
3618 reflections	(Δ/σ)_max_ = 0.006
175 parameters	Δρ_max_ = 0.30 e Å^−^^3^
3 restraints	Δρ_min_ = −0.35 e Å^−^^3^

### Special details

**Table:**

Geometry. Bond distances, angles *etc*. have been calculated using the rounded fractional coordinates. All su's are estimated from the variances of the (full) variance-covariance matrix. The cell e.s.d.'s are taken into account in the estimation of distances, angles and torsion angles
Refinement. Refinement on *F*^2^ for ALL reflections except those flagged by the user for potential systematic errors. Weighted *R*-factors *wR* and all goodnesses of fit *S* are based on *F*^2^, conventional *R*-factors *R* are based on *F*, with *F* set to zero for negative *F*^2^. The observed criterion of *F*^2^ > σ(*F*^2^) is used only for calculating -*R*-factor-obs *etc*. and is not relevant to the choice of reflections for refinement. *R*-factors based on *F*^2^ are statistically about twice as large as those based on *F*, and *R*-factors based on ALL data will be even larger.

### Fractional atomic coordinates and isotropic or equivalent isotropic displacement parameters (Å^2^)

**Table:**

	*x*	*y*	*z*	*U*_iso_*/*U*_eq_	
Si1	0.53721 (14)	0.18235 (12)	0.70600 (11)	0.0684 (3)	
N1	0.6155 (3)	0.3381 (3)	0.8362 (3)	0.0570 (7)	
N2	0.7515 (3)	0.3069 (3)	0.9818 (3)	0.0624 (8)	
C1	0.5921 (4)	0.3128 (4)	1.0536 (4)	0.0562 (9)	
C2	0.5185 (5)	0.3059 (4)	1.1879 (4)	0.0669 (10)	
H2	0.5760	0.2927	1.2499	0.080*	
C3	0.3555 (5)	0.3197 (4)	1.2249 (4)	0.0722 (11)	
H3	0.3016	0.3143	1.3147	0.087*	
C4	0.2689 (5)	0.3415 (4)	1.1314 (4)	0.0673 (10)	
H4	0.1590	0.3509	1.1604	0.081*	
C5	0.3419 (4)	0.3495 (4)	0.9978 (4)	0.0625 (9)	
H5	0.2844	0.3649	0.9353	0.075*	
C6	0.5059 (4)	0.3332 (4)	0.9609 (3)	0.0546 (8)	
C7	0.7597 (4)	0.3221 (4)	0.8533 (4)	0.0634 (10)	
H7	0.8535	0.3216	0.7846	0.076*	
C8	0.5773 (5)	0.3529 (4)	0.7092 (3)	0.0634 (10)	
H8A	0.6649	0.3954	0.6294	0.076*	
H8B	0.4847	0.4153	0.7007	0.076*	
C9	0.7201 (6)	0.0716 (5)	0.7079 (6)	0.1093 (17)	
H9A	0.7050	−0.0136	0.7007	0.164*	
H9B	0.7453	0.0508	0.7931	0.164*	
H9C	0.8055	0.1197	0.6310	0.164*	
C10	0.4835 (6)	0.2278 (6)	0.5440 (5)	0.1070 (17)	
H10A	0.3868	0.2840	0.5472	0.160*	
H10B	0.4690	0.1442	0.5337	0.160*	
H10C	0.5666	0.2790	0.4667	0.160*	
C11	0.3721 (6)	0.0963 (5)	0.8586 (5)	0.1049 (16)	
H11A	0.3672	0.0022	0.8668	0.157*	
H11B	0.2741	0.1463	0.8470	0.157*	
H11C	0.3897	0.0958	0.9409	0.157*	
C12	0.8871 (5)	0.2883 (5)	1.0387 (4)	0.0781 (12)	
H12A	0.8701	0.3488	1.0902	0.094*	
H12B	0.9831	0.3152	0.9629	0.094*	
C13	0.9078 (5)	0.1417 (5)	1.1316 (6)	0.0889 (14)	
H13	0.9301	0.0731	1.0937	0.107*	
C14	0.8967 (6)	0.1037 (6)	1.2611 (7)	0.1190 (19)	
H14A	0.8744	0.1698	1.3021	0.143*	
H14B	0.9109	0.0101	1.3139	0.143*	
O1W	1.0389 (5)	0.3039 (4)	0.5704 (4)	0.1283 (12)	
H1W	1.0104	0.3435	0.4962	0.192*	
H2W	1.0969	0.3573	0.5761	0.192*	
Br1	0.85464 (5)	0.48204 (5)	0.31301 (5)	0.0895 (2)	

### Atomic displacement parameters (Å^2^)

**Table:**

	*U*^11^	*U*^22^	*U*^33^	*U*^12^	*U*^13^	*U*^23^
Si1	0.0770 (8)	0.0756 (7)	0.0588 (6)	−0.0017 (6)	−0.0259 (6)	−0.0264 (6)
N1	0.0539 (18)	0.067 (2)	0.0517 (17)	−0.0005 (14)	−0.0141 (14)	−0.0253 (15)
N2	0.0522 (18)	0.076 (2)	0.065 (2)	−0.0021 (15)	−0.0165 (15)	−0.0321 (17)
C1	0.052 (2)	0.064 (2)	0.055 (2)	−0.0033 (17)	−0.0155 (17)	−0.0241 (18)
C2	0.071 (3)	0.080 (3)	0.055 (2)	−0.005 (2)	−0.018 (2)	−0.029 (2)
C3	0.072 (3)	0.082 (3)	0.059 (2)	−0.007 (2)	−0.005 (2)	−0.030 (2)
C4	0.054 (2)	0.073 (3)	0.072 (3)	−0.0029 (19)	−0.008 (2)	−0.031 (2)
C5	0.055 (2)	0.066 (2)	0.066 (2)	0.0009 (18)	−0.0174 (19)	−0.0259 (19)
C6	0.052 (2)	0.061 (2)	0.053 (2)	−0.0012 (16)	−0.0122 (17)	−0.0258 (17)
C7	0.053 (2)	0.076 (3)	0.062 (2)	−0.0019 (18)	−0.0085 (18)	−0.032 (2)
C8	0.069 (2)	0.072 (3)	0.045 (2)	−0.0009 (19)	−0.0158 (18)	−0.0188 (18)
C9	0.107 (4)	0.087 (3)	0.137 (5)	0.015 (3)	−0.043 (3)	−0.044 (3)
C10	0.121 (4)	0.144 (5)	0.079 (3)	−0.010 (3)	−0.041 (3)	−0.053 (3)
C11	0.120 (4)	0.103 (4)	0.090 (3)	−0.039 (3)	−0.011 (3)	−0.035 (3)
C12	0.056 (2)	0.107 (4)	0.085 (3)	−0.005 (2)	−0.027 (2)	−0.042 (3)
C13	0.067 (3)	0.099 (4)	0.115 (4)	0.009 (2)	−0.044 (3)	−0.044 (3)
C14	0.098 (4)	0.117 (5)	0.130 (5)	0.009 (3)	−0.055 (4)	−0.022 (4)
O1W	0.124 (3)	0.130 (3)	0.132 (3)	−0.002 (2)	−0.047 (2)	−0.042 (2)
Br1	0.0699 (3)	0.1215 (4)	0.0769 (3)	0.0007 (3)	−0.0195 (2)	−0.0391 (3)

### Geometric parameters (Å, °)

**Table:**

Si1—C9	1.840 (5)	C8—H8A	0.9700
Si1—C10	1.842 (4)	C8—H8B	0.9700
Si1—C11	1.854 (5)	C9—H9A	0.9600
Si1—C8	1.893 (4)	C9—H9B	0.9600
N1—C7	1.329 (4)	C9—H9C	0.9600
N1—C6	1.390 (4)	C10—H10A	0.9600
N1—C8	1.478 (4)	C10—H10B	0.9600
N2—C7	1.330 (4)	C10—H10C	0.9600
N2—C1	1.392 (4)	C11—H11A	0.9600
N2—C12	1.480 (5)	C11—H11B	0.9600
C1—C2	1.383 (5)	C11—H11C	0.9600
C1—C6	1.390 (5)	C12—C13	1.486 (6)
C2—C3	1.377 (5)	C12—H12A	0.9700
C2—H2	0.9300	C12—H12B	0.9700
C3—C4	1.397 (5)	C13—C14	1.285 (7)
C3—H3	0.9300	C13—H13	0.9300
C4—C5	1.372 (5)	C14—H14A	0.9300
C4—H4	0.9300	C14—H14B	0.9300
C5—C6	1.387 (5)	O1W—H1W	0.8512
C5—H5	0.9300	O1W—H2W	0.8513
C7—H7	0.9300		
			
C9—Si1—C10	111.7 (2)	Si1—C8—H8A	108.8
C9—Si1—C11	110.6 (2)	N1—C8—H8B	108.8
C10—Si1—C11	110.6 (2)	Si1—C8—H8B	108.8
C9—Si1—C8	107.8 (2)	H8A—C8—H8B	107.7
C10—Si1—C8	105.9 (2)	Si1—C9—H9A	109.5
C11—Si1—C8	110.15 (19)	Si1—C9—H9B	109.5
C7—N1—C6	108.0 (3)	H9A—C9—H9B	109.5
C7—N1—C8	126.3 (3)	Si1—C9—H9C	109.5
C6—N1—C8	125.7 (3)	H9A—C9—H9C	109.5
C7—N2—C1	107.9 (3)	H9B—C9—H9C	109.5
C7—N2—C12	126.3 (3)	Si1—C10—H10A	109.5
C1—N2—C12	125.8 (3)	Si1—C10—H10B	109.5
C2—C1—C6	121.7 (3)	H10A—C10—H10B	109.5
C2—C1—N2	131.6 (3)	Si1—C10—H10C	109.5
C6—C1—N2	106.7 (3)	H10A—C10—H10C	109.5
C3—C2—C1	116.5 (3)	H10B—C10—H10C	109.5
C3—C2—H2	121.8	Si1—C11—H11A	109.5
C1—C2—H2	121.8	Si1—C11—H11B	109.5
C2—C3—C4	121.9 (4)	H11A—C11—H11B	109.5
C2—C3—H3	119.0	Si1—C11—H11C	109.5
C4—C3—H3	119.0	H11A—C11—H11C	109.5
C5—C4—C3	121.7 (4)	H11B—C11—H11C	109.5
C5—C4—H4	119.2	N2—C12—C13	111.7 (3)
C3—C4—H4	119.2	N2—C12—H12A	109.3
C4—C5—C6	116.6 (3)	C13—C12—H12A	109.3
C4—C5—H5	121.7	N2—C12—H12B	109.3
C6—C5—H5	121.7	C13—C12—H12B	109.3
C5—C6—N1	131.6 (3)	H12A—C12—H12B	107.9
C5—C6—C1	121.7 (3)	C14—C13—C12	124.1 (5)
N1—C6—C1	106.7 (3)	C14—C13—H13	118.0
N2—C7—N1	110.8 (3)	C12—C13—H13	118.0
N2—C7—H7	124.6	C13—C14—H14A	120.0
N1—C7—H7	124.6	C13—C14—H14B	120.0
N1—C8—Si1	113.9 (2)	H14A—C14—H14B	120.0
N1—C8—H8A	108.8	H1W—O1W—H2W	109.9

### Hydrogen-bond geometry (Å, °)

**Table:**

*D*—H···*A*	*D*—H	H···*A*	*D*···*A*	*D*—H···*A*
C7—H7···O1W	0.93	2.46	3.351 (5)	161.
O1W—H1W···Br1	0.85	2.62	3.445 (4)	165.
O1W—H2W···Br1^i^	0.85	2.59	3.360 (4)	152.

Symmetry codes: (i) −*x*+2, −*y*+1, −*z*+1.

### Table 2 π-π contacts (Å, °)

Cg1:  N1,N2,C1,C6,C7; Cg2:  C1,C2,C3,C4,C5,C6; ccd: Distance between ring centroids; ipd: mean interplanar distance (Distance from one plane to the neighbouring centroid); sa: mean slippage angle (Angle subtended by the intercentroid vector to the plane normal). For details, see Janiak (2000).

**Table:**

ring 1/ring 2	ccd	ipd	sa
Cg1->Cg2^ii^	3.521 (3)	3.386	15.9
Cg2->Cg2^ii^	3.575 (2)	3.396	18.2

Symmetry code: (ii) 1-x,1-y,2-z.
